# Proteomic analysis of the abdominal aortic aneurysm wall

**DOI:** 10.1007/s00595-012-0480-6

**Published:** 2013-03-22

**Authors:** Jiri Molacek, Jan Mares, Vladislav Treska, Karel Houdek, Jan Baxa

**Affiliations:** 1 School of Medicine in Pilsen, Charles University in Prague, Husova 3, 306 05 Pilsen, Czech Republic; 2 Vascular Surgery Department, University Hospital in Pilsen, Alej Svobody 80, 304 60 Pilsen, Czech Republic; 3 Proteomic Laboratory, University Hospital in Pilsen, Alej Svobody 80, 304 60 Pilsen, Czech Republic; 4 Department of Imagine Techniques, University Hospital in Pilsen, Alej Svobody 80, 304 60 Pilsen, Czech Republic

**Keywords:** Abdominal aortic aneurysm, Ethiology of AAA, Proteomic analysis

## Abstract

**Purposes:**

A ruptured AAA (rAAA) is a common cause of death in males over 60 years of age, and the global mortality from rAAA exceeds 80 %. The pathological processes occurring in the wall of the developing AAA are still unclear. The potential pathophysiological mechanisms underlying aortic aneurysms have been examined by many studies using immunohistochemistry and were, therefore, targeted at specific, preselected protein antigens.

**Methods:**

We collected samples of tissue from anterior wall of an aneurysm sac from 15 patients indicated for AAA resection (group A) during the period from 2010 to 2011. These samples were subjected to a proteomic analysis. In addition, we collected control samples of identical aortic tissue from 10 heart-beating deceased organ donors (group B).

**Results:**

A total of 417 differentially expressed protein fractions were identified, 18 of which were only detected in the healthy controls, while 85 were specific for aneurysm tissue and 314 were detectable in both groups. In 175 protein fractions, the gel-derived spot volumes differed significantly between aneurismal and healthy aortic tissue.

**Conclusions:**

We found a significant difference in the proteome of the AAA tissue and non-dilated aortic tissue. We demonstrated that the AAA proteome is considerably richer and more varied than the healthy and atherosclerotic aorta. We believe that our results clearly demonstrate a completely different etiopathogenesis of atherosclerosis and aneurismal disease.

## Introduction

A rupturing abdominal aortic aneurysm (AAA) is still a common cause of death in males over 60 years of age, and the global mortality from ruptured subrenal abdominal aortic aneurysms exceeds 80 %. The pathological processes occurring in the wall of the developing AAA are still unclear. The atherosclerotic theory was abandoned many years ago, and it is apparent that multiple factors are involved in the pathogenesis. The role of inflammation in the aortic wall and degradation of the extracellular matrix have been discussed, but even the theory of infectious etiology has not been completely abandoned. Clarification of the pathomechanism(s) would markedly facilitate the prevention or treatment of an AAA. A determination of the proteins in the AAA wall and its comparison with the healthy, non-dilated aortic wall has not been previously reported.

The pathophysiological mechanisms underlying aortic aneurysms have been examined by many studies using immunohistochemistry and, therefore, have targeted specific, preselected protein antigens [1–3]. However, during the last decade, molecular technologies, including mass spectrometry-based proteomics, have become widely available, offering an unbiased approach to analyze complex protein mixtures. In principle, a proteomic analysis allows for the identification of all relevant proteins (e.g. reaching significant differences in abundance in two groups) within a system, without any pre-existing knowledge of these proteins.

Most of the past proteomic studies have focused on tissue cultures of several mesenchymal elements obtained from aneurysms [4]. This represents the most convenient model for proteomic studies, because it generates very homogenous and reproducible material with virtually no contamination. However, the development of aneurysms is undoubtedly governed not only by inherited predispositions, but also by various epigenetic and environmental factors. Indeed, many regulatory mechanisms involved in aneurysm formation, including those of transcription and further downstream, are probably lost during cell culture. Moreover, the cells transferred into a culture medium are exposed to a completely new environment, and during subsequent proliferation and maturation, can easily lose their in vivo characteristics.

While such models can offer a good initial approximation of the processes responsible for the development of aneurysms, the goal should remain the analysis of raw clinical samples. Such an approach would be capable of capturing more subtle changes in the extracellular matrix and the cells’ proteome accumulated in the aortic wall during the genesis of the aneurysm. However, such studies are extremely challenging in terms of achieving maximum sample purity. The sample procurement must follow the most stringent principles; otherwise, any further analysis would be hampered by variable contamination with blood and other tissues. Obviously, the harvesting of samples must not interfere with the surgical procedure; nor can it be delayed due to risk of autolysis. These preconditions necessitate defining a standard algorithm that is simple and clear cut enough to be feasible, while still delivering reproducible results.

The aim of this study was to implement a technique for harvesting representative aortic aneurysm wall specimens to verify their applicability for proteomic analysis and to possibly identify novel biomarkers or pathophysiological mechanisms for AAAs. Our objective was to conduct a proteomic analysis of the abdominal aortic aneurysm wall and to compare the findings with those of non-dilated wall tissue, i.e. a relatively “healthy” aorta obtained during organ removal from heart-beating deceased organ donors. In this control group, however, some degree of atherosclerosis of the aortic wall would be expected. Hence, it was considered to be impossible that a completely healthy aortic wall would be obtained from subjects in this second group.

## Methods

**Fig. 1:**
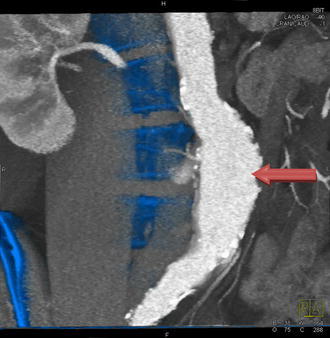
CT angiography of AAA. *Arrow* the site of sample collection from the AAA wall

We collected samples of tissue from the aneurysm sac from the anterior wall of an AAA (Fig. 1) in 15 patients indicated for AAA resection (group A) in the Department of Surgery, University Hospital in Plzeň, during the period from 2010 to 2011. This sample was subjected to a proteomic analysis (see description below). In addition, we collected control samples of identical aortic tissue from 10 heart-beating deceased organ donors (group B).

The entire project was approved by the Ethics Committee of the Faculty of Medicine of Plzeň, Charles University in Prague. Patients in group A provided informed consent for the use of their aneurysm tissue specimens for scientific purposes.

### Inclusion criteria and harvesting methods

Patients with subrenal abdominal aortic aneurysms, in whom an elective resection procedure was indicated, were enrolled in group A of the study. According to the available evaluations and tests, patients with a post-traumatic etiology of the AAA were ruled out (as were patients in whom the aneurysm had not developed based on aortic dissection), and no fungal or inflammatory etiology of the AAA was suspected in any of the patients. The aneurysm did not reach beyond the level of the origin of the renal arteries, and all of the patients had a normal width of the thoracic aorta. All cases involved asymptomatic AAA, without signs of leaking or rupture. Patients with excessively calcified atherosclerotic changes in the aortic wall were excluded from the study.

The patients had no severe comorbidities, such as diabetes, malignant disease or any other systemic disease that could modify the results of the analysis. No acute infectious disease was present in any of the patients. The operation (resection of the AAA and aortic replacement using an artificial vascular prosthesis) was always performed by a single surgeon, a member of the research team, who was fully informed about the exact procedure for tissue collection. All operations were carried out in general anesthesia under standard conditions without any deviations from the commonly performed resections of the AAA (usual anesthetic agents, prophylactic antibiotics, 2 × 1 Vulmizolin GIV, Heparin 100 IU/kg). The tissue from the aneurysm wall was clipped with scissors from the anterior wall (approximately 1 cm^2^), without using electrocoagulation cautery. An identical part of the anterior wall, which is usually subject to resection, was always collected.

The patients enrolled in group B were heart-beating deceased organ donors for transplantation purposes, and the tissue sampling was again performed by a single surgeon. Tissue sampling was performed after the usual flushing of the kidneys and their explantation from the front surface of the non-dilated aorta (again approximately 1 cm^2^).

If a clear sclerotic plaque was present at the site of collection (in both groups) on the tunica intima, such plaque was removed, along with the adjacent fatty and fibrous tissue on the tunica adventitia. The tissue sample was immediately washed in normal saline and frozen in liquid nitrogen.

The proteomic analysis was performed in the proteomic laboratory of the Department of Internal Medicine, University Hospital in Plzeň.

### Sample preparation and solubilization

Aortic tissue samples were frozen in liquid nitrogen immediately after procurement, and then kept at −80 °C until final processing. During the analysis, the samples were first washed thrice with PBS (not allowed to melt), then ground in liquid nitrogen and extracted in a buffer containing detergent. Afterwards, the lysate was spun at 10000×*g* for 10 min at 4 °C to obtain the supernatant. The protein content was determined by the Bradford dye binding assay.

### Two-dimensional electrophoresis (2-DE)

Urea, CHAPS, Tris base, thiourea, sodium dodecylsulphate (SDS), dithiotreitol DTT, iodacetamide IAA (iodacetamide) and bromophenol blue used during the preparation of samples were purchased from Sigma (Sigma-Aldrich, Steinheim, Germany); immobilized pH gradient IPG buffer (ZOOM carrier Ampholytes 3–10) was purchased from Invitrogen (Invitrogen Corporation, Carlsbad, CA, USA). Equal amounts (200 μg) of protein in both the eluate and plasma samples were mixed with rehydration buffer (7 M urea, 4 % CHAPS, 40 mM Tris base, 2 M Thiourea, 2 % IPG buffer pH 3–10, 120 mM DTT, and a trace amount of bromophenol blue) to obtain a final volume of 185 ml. Samples were then rehydrated in IPG strips (11 cm, pH range 3–10 nonlinear, Bio-Rad), and focused in a Protean IEF cell (Bio-Rad, Hercules, USA). The IPG strips were rehydrated actively for 10 h at 50 V, followed by a stepwise isoelectric focusing (IEF) as follows: 250 V for 15 min, rapid ramp to 8000 V for 150 min, and finally 8000 V for the other 35,000 Vh. After isoelectric focusing, the IPG strips were equilibrated in equilibration buffer 1 (112 mM Tris base, 6 M urea, 30 % v/v glycerol, 4 % w/v SDS, 130 mM DTT and a trace of bromophenol blue) for 10 min, and subsequently alkylated in buffer 2 (112 mM Tris base, 6 M urea, 30 % v/v glycerol, 4 % w/v SDS, 135 mM IAA and a trace of bromophenol blue) for 10 min. Each equilibrated IPG strip was placed on the top of a 12.5 % criterion TRIS–HCl gel (Bio-Rad) and covered with 0.5 % agarose. Second-dimension separation was performed at 200 V until the bromophenol blue dye front reached the bottom of the gel. At the end of each run, the 2D gels were stained with Simply Blue stain (Invitrogen), and scanned with an Epson Perfection 4990 Photo scanner (Epson, Long Beach, CA, USA) at 400 dpi resolution and in 16 bit greyscale.

### The 2-DE pattern analysis and statistical analyses

A computer-aided analysis of 2-DE gel images was carried out using the PDQuest 2-D software program version 8.1 (Bio-Rad). A synthetic image was constructed out of the triplicate gels processed from each sample, using only spots consistently present in at least two gels. The protein quantity was determined relative to the integrated spot density. For subsequent studies of the characteristics of the protein distribution, only spots detectable in at least 50 % of patients were considered to be eligible.

The statistical analysis was carried out by means of the Statistica data analysis software program (Version 8.0, Statsoft Inc., Tulsa, OK, USA). The inter-gel variability was determined based on the coefficients of variation (CV) of relative spot intensities across triplicate experiments. An exploratory analysis was performed on the proteomic data to reduce their dimensionality before picking spots for identification. A principal component analysis (based on the correlation matrix of normalized protein intensities) and partial least squares–discriminant analysis were applied. The Mann–Whitney rank sum test was employed to compare non-dependent continuous variables. If not stated otherwise, data are given as medians (inter-quartile ranges). Differences were considered to be statistically significant for values of *p* < 0.05.

### In-gel tryptic digestion

Acetonitrile (ACN), ammonium bicarbonate, DTT, IAA, trifluoroacetic acid (TFA), formic acid and α-cyano-4-hydroxycinnamic acid (CHCA) were purchased from Sigma. First, selected spots were excised by an EXQuest spot cutter (Bio-Rad). The Simply Blue stain was removed by washing with 50 mM ammonium bicarbonate and ACN. Proteins in the gels were reduced with 10 mM DTT/50 mM ammonium bicarbonate at 56 °C for 45 min, and were alkylated with 55 mM IAA/50 mM ammonium bicarbonate (for 30 min, in the dark at room temperature). Gel plugs were washed with 50 mM ammonium bicarbonate and ACN, and dried by a SpeedVac. The dried gel particles were rehydrated with digestion buffer containing 12.5 ng/μl sequencing grade trypsin (Roche) in 50 mM ammonium bicarbonate at 4 °C. After 45 min, the remaining solution was removed, and was replaced with 50 mM ammonium bicarbonate. Tryptic digestion was performed overnight (37 °C). After digestion, the proteolytic peptides were subsequently extracted with 50 % ACN/25 mM ammonium bicarbonate, 5 % formic acid and 50 % ACN/H_2_0. The three extracts were pooled, and a 10 mM DTT solution in 50 mM ammonium bicarbonate was added. The mixture was then dried in a SpeedVac, and the resulting tryptic peptides were dissolved in 5 % formic acid solution and desalted using a ZipTip μC18 column (Millipore, Bedford, MA, USA).

### Matrix-assisted laser desorption/ionization (MALDI) time-of-flight (TOF) tandem mass spectrometry and protein identification

The proteolytic peptides were mixed with CHCA matrix solution (5 mg/mL CHCA in 0.1 % TFA/50 % ACN 1:1, v/v) at a 1:1 ratio, and 0.8 μl of this mixture was spotted onto MALDI targets. All mass spectra were acquired in a reflectron mode with a 4800 MALDI-TOF/TOF Analyzer (Applied Biosystems, Framingham, MA, USA). A total of 2000 and 3000 laser shots were acquired, and averaged to yield MS and MS/MS spectra, respectively. The MS/MS analyses were performed using a collision energy of 1 keV and collision gas pressure of 1.3e10^−6^ Torr. The MS peaks with an S/N above 15 were listed, and the ten strongest precursors with an S/N above 50 among the MS peaks were automatically selected for MS/MS acquisition. A mass filter was used to exclude autolytic peptides of trypsin and peaks of contaminant from the polypropylene tubes [5].

The resulting data were analyzed with the GPS Explorer™ 3.6 (Applied Biosystems) software program. Proteins were identified by searching against the human subset of the Swissprot protein database (release 54.6; December 4, 2007) using the MASCOT 2.1.0 search algorithm (Matrix Science, London, UK). The general parameters for peptide mass fingerprinting (PMF) searches were considered to allow for a maximum of two missed cleavages, ±50 ppm of peptide mass tolerance, variable methionine oxidation and fixed cysteine carbamidomethylation. The probability-based MOWSE scores were estimated by comparison of the search results with the estimated random match population, and were reported as −10log_10_(*p*), where *p* is the absolute probability. MOWSE scores greater than 55 were considered to be significant (*p* < 0.05) for PMF. A peptide charge state of +1 and fragment mass tolerance of ±0.25 Da were used for the MS/MS ion search. Individual MS/MS ions scores >28 indicated identity or extensive homology (*p* < 0.05) for the MS/MS ion search.

### Statistical analysis

All results were processed statistically (*T* test, Wilcoxon test, ANOVA), and evaluated by a statistician. *p* values <0.05 were considered to be statistically significant.

## Results

In the 15 patients in group A, the average age was 68.0 years (range 56–77 years). The average aneurysm diameter was 6.3 cm (range 5–9.5 cm). All aneurysms were asymptomatic. Samples were harvested during a planned aneurysm resection. Table 1 shows basic information about the patients in group A (age and six of the patient, and the maximum aneurysm diameter).

**Table 1:** Characteristics of the patients in group A

Patient no.	Age	Max. diameter of AAA (mm)	Sex
1	73	55	M
2	67	65	M
3	75	60	F
4	70	95	M
5	77	75	F
6	63	60	M
7	56	60	M
8	70	71	M
9	62	63	M
10	69	68	M
11	59	58	M
12	64	80	M
13	71	50	F
14	68	65	M
15	63	72	M

In the 10 patients in group B, the average age was 45.7 years (range 18–61 years). Five samples had moderate signs of atherosclerosis (a thicker tunica intima), two samples were without any pathological signs (young patients, aged 18 and 25 years old), and three patients had severe signs of atherosclerosis. Table 2 shows the basic information for the patients in group B (age, sex, cause of death and grade of atherosclerosis).

**Table 2:** Characteristics of the patients in group B

Patient no.	Age	Sex	Cause of brain death	Grade of atherosclerosis
16	25	M	Non-traumatic subarach. Hemorrhage	0
17	54	M	Non-traumatic subarach. Hemorrhage	**
18	47	M	Cranial trauma	**
19	18	M	Meningoencephalitis	0
20	41	F	Cranial trauma	**
21	36	M	Non-traumatic subarach. Hemorrhage	**
22	57	F	Cranial trauma	**
23	61	M	Cranial trauma	***
24	60	M	Non-traumatic subarach. Hemorrhage	***
25	58	F	Cranial trauma	***

*0* no signs of atherosclerosis, ** moderate signs of atherosclerosis (thick tunica intima, no calcification), *** severe signs of atherosclerosis (mild calcification)

The average aortic specimen weight was 111.1 (34.5) mg in group A and 86.4 (8.3) mg in group B. The first yielded 3.6 (1.0) mg of soluble protein (3.6 %) while from the latter group, 2.7 mg was retrieved (3.1 %). Soluble proteins were extracted from tissue lysates and resolved by 2-D gel electrophoresis. In this way, 417 proteins fractions were identified, 18 of which were specific to healthy controls (group B), 85 were specific to aneurysm tissue (group A) and 314 were detectable in both groups, but were differentially expressed. In 175 protein fractions, the gel-derived spot volumes differed significantly between the aneurismal and healthy aortic tissue. Out of these, the hundred most abundant and best-resolved spots were picked for identification by mass spectrometry, which was successfully accomplished for 25 proteins (Table 3).

**Table 3:** The 25 identified proteins differentially expressed between aneurysm and normal tissue

Name	Function	SwissProt entry	Spot volume (aneurysm/control)	*p*
Alpha-1-antitrypsin	Serine protease inhibitor (plasma)	A1AT_HUMAN	2.262844	<0.05
Alpha-cardiac actin	Cytoskeleton, contractility	ACTC_HUMAN	0.362874	<0.001
Alcohol dehydrogenase 1B	Enzyme, metabolism	ADH1B_HUMAN	0.405067	<0.01
Albumin	Oncotic agent (plasma)	ALBU_HUMAN	2.671886	<0.01
Annexin A2	Plasminogen receptor, fibrinolysis	ANXA2_HUMAN	0.422765	<0.05
Annexin A5	Thromboplastin inhibitor, anticoagulation	ANXA5_HUMAN	0.291819	<0.01
Collagen alpha-2 (VI)	Muscle constituent	CO6A2_HUMAN	1.890603	<0.05
Cysteine and glycine-rich protein 1	Unknown	CSRP1_HUMAN	0.337667	<0.05
Destrin	Actin depolymerization	DEST_HUMAN	0.255535	<0.001
Destrin	Actin depolymerization	DEST_HUMAN	0.623054	<0.01
Alpha-enolase	Plasminogen receptor, fibrinolysis	ENOA_HUMAN	0.486065	<0.01
Endoplasmin	Chaperon	ENPL_HUMAN	1.544984	<0.05
Fibrinogen gamma	Coagulation factor (plasma)	FIBG_HUMAN	2.618752	<0.05
Glyceraldehyde-3-phosphate dehydrogenase	Glycolysis, nuclear functions (transcriprion)	G3P_HUMAN	0.429752	<0.001
Heat shock 70 kDa protein	Chaperon	HSP71_HUMAN	0.177951	<0.001
Ig alpha-2	Immunoglobulin (plasma)	IGHA2_HUMAN	38.39577	<0.05
Ig gamma-1	Immunoglobulin (plasma)	IGHG1_HUMAN	3.64375	<0.01
Keratin, type II cytoskeletal 1	Kinase activity regulator	K2C1_HUMAN	0.478847	<0.001
Pre-mRNA-processing factor 17	Splicing	PRP17_HUMAN	3.445882	<0.01
Transgelin	Actin crosslinking	TAGL_HUMAN	0.274982	<0.01
Protein-glutamine gamma-glutamyltransferase 2	Protein crosslinking	TGM2_HUMAN	0.232779	<0.01
Transferrin	Transport (plasma)	TRFE_HUMAN	2.948914	<0.01
Transferrin	Transport (plasma)	TRFE_HUMAN	2.522563	<0.05
Vimentin	Filaments (fibroblasts)	VIME_HUMAN	0.626753	<0.05
Vimentin	Filaments (fibroblasts)	VIME_HUMAN	0.70918	<0.01

**Fig. 2:**
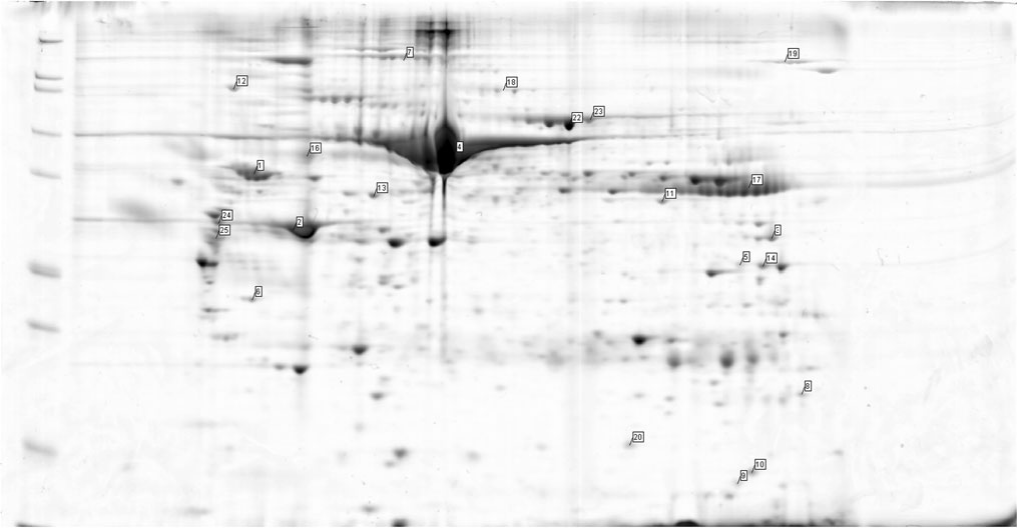
Examples of the spectrometric findings on the gels from group A—patient no. 1 (aneurysm wall)

**Fig. 3:**
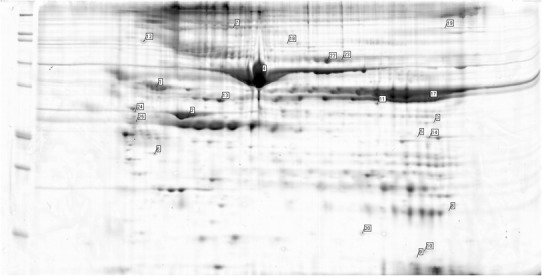
Examples of the spectrometric findings on the gels from group A—patient no. 2 (aneurysm wall)

**Fig. 4:**
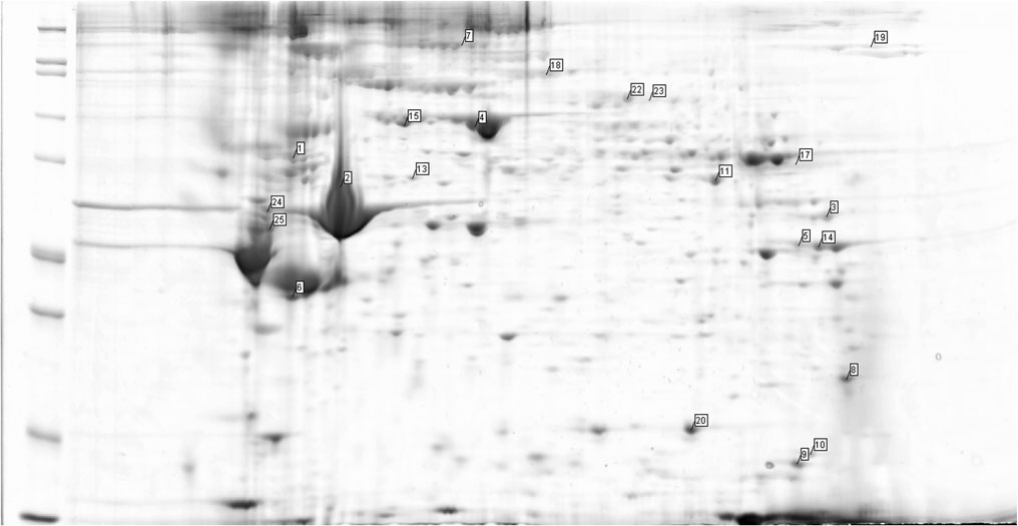
Examples of the spectrometric findings on the gels from group B—patient no. 16 (healthy aorta)

**Fig. 5:**
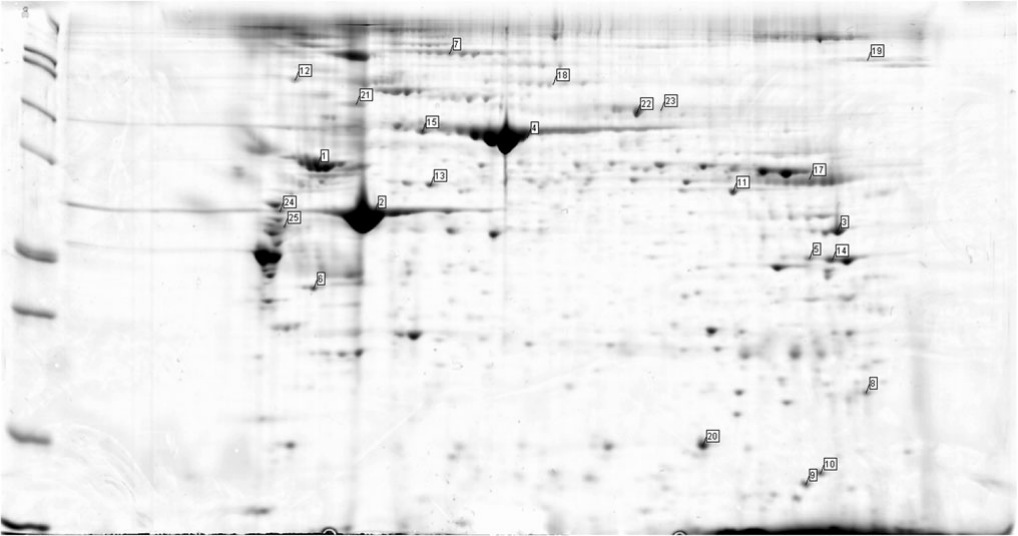
Examples of the spectrometric findings on the gels from group B—patient no. 17 (moderate signs of atherosclerosis)

For several proteins, the finding of discrepant expression in the aneurismal and healthy corresponding atherosclerotic aortic wall was in accordance with previous reports (the differential expression was detected various methods), and included alpha-1-antitrypsin [6], alcohol dehydrogenase [7], annexins [8, 9] and glyceraldehyde-3-phosphate dehydrogenase [9]. In other cases, an association of the specific protein with aneurysm formation is biologically plausible, especially in the case of extracellular matrix and cytoskeleton components. These newly identified proteins that may be involved in aneurysm formation include alpha actin, collagen VI, type II keratin, vimentin, destrin, transgelin, protein-glutamine gamma-glutamyltransferase 2 and alpha-enolase. The role of some of the other identified proteins in aneurysm development seems conceivable; however, the exact mechanism is unclear and there exists a possibility that their detection may be due to variable contamination. These included mainly chaperones and regulatory proteins, including heat shock 70 kDa protein, endoplasmin and pre-mRNA-processing factor 17. We also identified albumin, immunoglobulin and transferring as being differentially expressed, but in the case of these high-abundance plasma proteins, their prevalence in aneurysm tissue may be attributable to its different morphology (i.e. the presence of an intraluminal thrombus) rather than functional properties. Examples of spectrometric findings on the gels from groups A and B can be seen in Figs. 2, 3, 4 and 5.

### Summary of the results

In the aneurysmal aortic tissue, we found a significantly higher expression of alpha-1-antitrypsin, albumin, collagen alpha-2 (VI), endoplasmin, fibrinogen gamma, Ig alpha-2, Ig gamma-1, Pre-mRNA-processing factor 17 and transferrin compared to the levels in the healthy aortas. In contrast, we found higher expression levels of alpha-cardiac actin, alcohol dehydrogenase 1B, annexin A2 a annexin A5, cysteine and glycine-rich protein 1 cysteine and glycine-rich protein 1, destrin, alpha-enolase, glyceraldehyde-3-phosphate dehydrogenase, heat shock 70 kDa protein, keratin, type II cytoskeletal 1, transgelin, protein-glutamine gamma-glutamyltransferase 2 and vimentin in the healthy aortic tissue compared to the AAA samples.

## Discussion

At present, a proteomic analysis is most frequently employed when searching for markers of malignant diseases [10–12]. The aim of such studies is to identify markers that can be used in clinical practice either as indicators of progression of malignancy, preferably as markers that are detectable prior to the clinical manifestation of the tumor itself. In some cases, these objectives are successfully met. In recent years, there have been efforts made to find similar markers of cardiovascular disease. The proteomic analysis has been most frequently used to understand the etiopathogenesis of atherosclerosis or abdominal aortic aneurysms. It would be optimal to identify an easily detectable marker that correlates with the progression of AAA or its changes in the wall that lead to a rupture. It would also be helpful to identify high-risk aneurysms, for which early surgical treatment would be indicated.

The very first results have already been published in this field [1, 2, 13, 14]. It is known that some AAA can rupture even with a smaller diameter (about 5 cm), while others are stable despite exceeding 8 cm in size. It is obvious that the aneurysm size is not the only factor determining the risk of rupture. Other factors have also been discussed, such as the presence of infection, progression of the inflammatory reaction in the wall, distensibility [15], etc. Precise knowledge of the structure of the wall can be helpful in identifying such factors.

One of the options for identifying these factors is a proteomic analysis. In the past, these analyses have often been performed using serum [16], which is advantageous due to the possibility of performing a minimally invasive screening that can be done for anyone. The most common method is a proteomic analysis of tissue cultures, which are technically easy, without any risk of contamination of the samples, but is also associated with its own limitations. For example, tissue cultures often do not reflect the real clinical conditions. In our opinion, determination of the proteome in the patient’s own tissue is more important for the precise analysis of the AAA wall. Of course, such an analysis is only possible after resection of the aneurysm, but the results may be crucial for identifying proteins that play a role in the etiopathogenesis and progression of AAA. Some authors have also analyzed the proteome of the intraluminal thrombus (ILT) for the same reason [17]. The proteomic analysis of various types of AAAs (asymptomatic, symptomatic, rapidly growing or ruptured) may also provide benefits. The first studies regarding this topic have already been published. [4]. In this case, the results may help to understand the pathogenesis of AAA progression.

In our present study, we found significant differences in the proteomes of the aortic aneurysm tissue and the non-dilated aortas with atherosclerosis. We also demonstrated that the AAA proteome is considerably richer and more varied than the healthy and atherosclerotic aortas. We believe that our results clearly demonstrate that there is a completely different etiopathogenesis of atherosclerosis and aneurismal disease. Therefore, AAAs should never be marked as atherosclerotic, although such labeling continues in the literature. We intentionally avoided studying specific pre-protein fractions, since our effort was to analyze the sample proteomes as comprehensively as possible. However, we also realize the potential errors of our analysis, and the contamination of samples with plasma was still a possibility, although we always washed the samples thoroughly with saline. Our results suggest that there may have been contamination with plasma proteins. Even this, however, can be understood as a potential marker for increased vulnerability of the AAA endothelium, confirming that endothelial “injury” may be one of the first steps in the pathogenesis of AAA.

The interpretation of our results is very complicated; more than 100 differentially expressed proteins were observed, and twenty-five of these were identified. While some of these may have been due to contamination, many others may be involved in the pathogenesis of AAAs. Indeed, a simple biomarker for the development or progression of AAA was not expected. However, we hope that this work will contribute to understanding the etiopathogenesis of AAA (in addition to the studies of the inflammatory response, matrix metalloproteinase activities, genomics and so on).

Our research must logically proceed toward the standardizing of our methods, not only in terms of the collection of samples, but also the presentation of the results. We propose that future studies that separate the individual layers of the aortic wall, mainly the tunica intima, from the rest of the wall, should be performed to gain a more detailed understanding of the proteomes of the different structures. That means that there will be a separate analysis of the tunica intima and the tunica adventitia (which is probably crucial in the pathogenesis of the aneurismal dilation). Using this technique, we have already started collecting new samples to be analyzed. The objective of this future study is to eliminate as many possible factors influencing the final analysis as can be accomplished. This undoubtedly will help us achieve more accurate results.

### Benefits for clinical practice

Our results, if correlated with the plasma levels of proteins in the future, could help to identify biomarkers of vascular lesions (atherosclerosis, aneurysms). These markers could then be used to diagnose metabolically active, and thus potentially risky, aneurysms.
